# Entropic Analysis of Geographical Subzones for the Maule *M_w_*8.8 (2010) Earthquake

**DOI:** 10.3390/e28070760

**Published:** 2026-07-02

**Authors:** Javiera Olave, Eugenio E. Vogel, Pablo Díaz, Denisse Pastén, Gonzalo Saravia

**Affiliations:** 1 Departamento de Ciencias Físicas, Universidad de La Frontera, Casilla 54-D, Temuco 4811230, Chile; j.olave01@ufromail.cl (J.O.); eugenio.vogel@ufrontera.cl (E.E.V.); pablo.diaz@ufrontera.cl (P.D.); 2 CEDENNA, FINARQ, Universidad Central de Chile, Av. Sta. Isabel 1186, Santiago 8330601, Chile; 3 Departamento de Física, Facultad de Ciencias, Universidad de Chile, Las Palmeras 3425, Ñuñoa 7800003, Chile; 4 Independent Researcher, Los Eucaliptus, 1189, Temuco 4812537, Chile; gonzalo.saravia@gmail.com

**Keywords:** Maule earthquake, Shannon entropy, Tsallis entropy, mutability

## Abstract

Three entropic functions are used to characterize the seismic activity prior and after the major $M_{w}$8.8 earthquake of Maule (Chile) dated on 27 February 2010. Shannon entropy, mutability, and Tsallis entropy are calculated globally on the seisms extracted from the catalog of the National Center of Seismology (Chile). Calculations are done both globally on the whole data and also dynamically on windows of different number of seisms after filtering data with a Gutenberg–Richter analysis. The data are time series based on two observables: seism magnitudes and inter-event intervals. It is found that the two entropies and mutability give similar descriptions on the different regimes present between 2005 and 2022. However, mutability can be calculated in a direct and straightforward way. It is also found that the results for inter-event intervals produce more contrast between periods than the corresponding ones for magnitudes. The region spanning six degrees in latitude is split in five overlapping subzones of two degrees each. This allows us to find the way the rupture takes place: from south to north, for about 400 km.

## 1. Introduction

One of the most challenging natural phenomena is the occurrence of great earthquakes. In this century, we can count almost seven megathrusts with magnitudes greater than $M_{w}$8.5 worldwide. All of them have occurred in the so-called Pacific Ring of Fire, an area whose tectonic plate dynamics are controlled by the subduction of one plate beneath another.

In this article, we will focus our attention on the large earthquake that occurred on the Pacific coast of Chile, in the subduction zone between the Nazca and South American plates, which was registered on 27 February 2010 off the Maule coast, with a magnitude 8.8 [1,2]. This earthquake broke a region near a zone where the last great earthquake of $M_{w}$8.5 occurred in 1835 in Concepción [3]. The zone where this megathrust occurred had a slip deficit of 12 m [4] and was fully coupled for years. This earthquake occurred in a region that encompasses parts of the rupture zones of three previous great earthquakes: the $M_{w}$8.3 earthquake of 21 May 1960 in Concepción, the $M_{w}$9.5 earthquake of 22 May 1960 in Valdivia, and the $M_{w}$8.5 earthquake of 16 August 1906 in Valparaíso, in the central zone of Chile [5]. This earthquake altered the geography of the region near Maule, causing devastation from both the earthquake itself and the accompanying tsunami. These characteristics of the large earthquake of Maule 2010 give us a valuable environment to analyze the seismicity of that zone from some years before that earthquake to April 2022, from the point of view of the entropy approach.

In this article, we apply an entropic approach to a seismic data set measured by the Centro Sismológico Nacional (CSN) [6] in the zone of the Maule earthquake, focusing our attention on the temporal evolution of three entropies: Shannon entropy [7,8,9], Tsallis entropy [10], and the entropy-related function mutability [11]. These three functions have shown to provide valuable information related to the dynamical processes of the earthquake occurrence and the seismicity after the earthquake occurrence [12,13,14,15,16,17,18,19,20]. Here, we study the $M_{w}$8.8 Maule earthquake of 2010 over time, separating the space from the north of the rupture zone to the south, and showing the effect of the large earthquake on the entropies along the subduction zone in central-southern Chile.

## 2. Seismic Data

**Figure 1:**
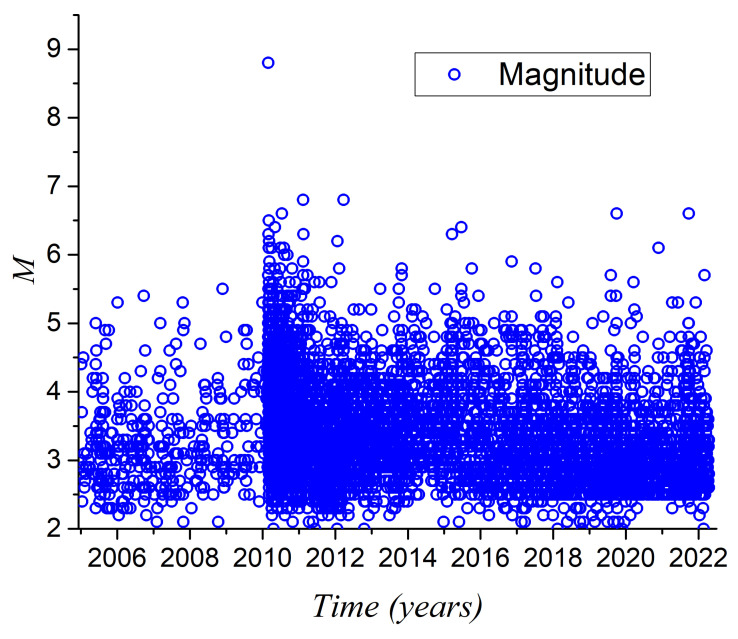
Sequence of magnitudes of the 17,147 seisms initially considered in this study. The different seismic density before and after the main earthquake is evident.

The seismic data analyzed in this paper are extracted from the catalog of the Centro Nacional de Sismología (CSN) of Chile [6]. We consider initially seisms with epicenter within [−32°,−38°] in latitude and [−70°,−76°] in longitude and up to 80 km depth. A total of 17,147 seisms were collected between 1 January 2005 and 12 April 2022. Although there was no magnitude threshold, very few seisms with magnitudes under 2.0 were present. The time evolution of their magnitudes is plotted in Figure 1.

**Figure 2:**
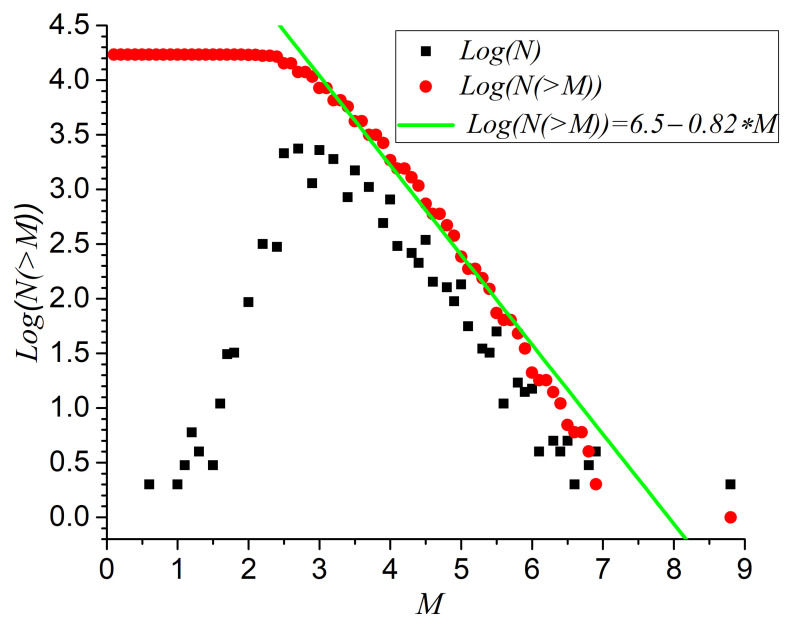
GR plot for the 17,147 seisms initially studied in this work. To compute the magnitude of completeness, we use the maximum curvature method (MAXC).

A histogram of magnitudes based on the data in Figure 1 yields the Gutenberg–Richter analysis, as shown in Figure 2. The lower curve gives the logarithm of the abundance N(M), while the upper curve gives the logarithm of the accumulative function $N(M^{\prime }>M)$, giving the logarithm of the sum of abundances with magnitudes $M^{\prime }$ which are larger than the value *M* of the abscissa. Actually, the value of this function on the left-hand limit is log(17,147) = 4.23.

In order to determine the magnitude of completeness, we use the maximum curvature method (MAXC) [21]. The lower curve reaches its maximum at a magnitude of 2.6, which means it is the completeness cut. Namely, from there to the right, the upper curve can be approximately adjusted by a straight line that is also plotted in this figure; its expression is given in the inset. Thus, the slope of this decay has a value −0.82, which tells of interconnected events as is usual in highly seismic zones. After discarding all seisms with M≤2.5, there remain 15,395 events, which is what we consider from now onward. Usually, the *b* value of the Gutenberg–Richter law reaches values around 1.0 [22,23]. This parameter has been widely studied as a seismic hazard indicator and has been related to the stress in a specific zone [24,25,26,27]. For example, a comprehensive study conducted in Central America found values of *b* ranging from 0.67 to 1.12 [28,29]. In this work, they found that values for inter-plate seismicity are around 0.85; this value is consistent with the value found in the present catalog.

## 3. Methodology

Entropic methods will characterize the Maule seismic zone. Prior to an earthquake, the underground rocks can break in many different possible ways, each one associated to a possible state of the complex system. The logarithm of the number of such states is a measure of the entropy of the system as an extension of the early proposal of Boltzmann referring to thermal systems [30]. We will briefly summarize the methods, beginning with the well-known Shannon entropy [8,9,11], then we continue with the mutability, which can be considered as a dynamic extension of the Shannon entropy [20,31], and finally we make use of the non-extensive Tsallis entropy to seismology [18,32].

**Shannon entropy.** Let us consider a set of ρ events each one characterized by a measurement of a property. The number of repetitions for the i-th different value, namely the frequency $f_{i}$, leads to a histogram. The addition of all $f_{i}$ values renders ρ, the total number of records. Then, the normalized probability of measuring the i-th value is simply given by:

(1) $$p_{i}=\frac{f_{i}}{\rho }\;.$$

An example of the way the probability for the i-th value for a sequence of seisms is given by the fourth and fifth column of Table 1 (to be explained below), which considers just a portion of 32 seisms around the earthquake of magnitude 6.8 that occurred on 1 August 2019 precisely within the region under study.

With these probabilities, we can readily express Shannon entropy as

(2) $$H=-\sum_{i}^{\rho^{*}}p_{i}\;ln\left(p_{i}\right)\;,$$

where $\rho^{*}$ is the number of different values of the magnitudes *M* within the ρ original measurements.

**Mutability.** We can easily obtain the information content of the ρ measured values if we get the histogram for Shannon entropy. By encoding the original information in a specific way, we can create a compressed file and recover the original information if needed. As shown in [33] and its references, the wlzip data compressor achieves this. The algorithm and its operation manual are freely offered upon request (eugenio.vogel@ufrontera.cl). In any case, the next part provides the logic, allowing interested readers to create their own algorithms, as they have done previously. To explain the way this algorithm works, we make use of Table 1. 

The first column of Table 1 enumerates the ρ seisms, and column II lists their magnitudes. The third column of Table 1 contains the different values and their repetitions throughout the position indexes that keep track of the sequence of seisms stored in the second column. This information also leads to the histogram (fourth column) needed for the probabilities (fifth column) used to calculate the Shannon entropy.

We very briefly review a few examples in Table 1. Let us start with the first value of column 2, namely 2.9; we write it at the beginning of the third column, followed by its distance to the origin of the original file in column 2, namely, 0 (distance to itself in this case). To the right, we list the next appearances of this same value by their positions’ indexes and repetitions in the following way: 8, 2 means that next time the value 2.9 appears in the second column, it is 8 positions below its last appearance, and a comma followed by the number 2 indicates it is repeated twice; the next time we find the value 2.9, is 10 positions below its last appearance; its last appearance is 12 positions below the last time. The next new or different value in the second column is 2.6, which is found at a distance of 1 from the origin, appearing five positions below, and then five positions below for the last time. In this way, we continue until we find the last different value in the second column, namely, 3.1, which appears only once, 30 positions below the origin. The detailed construction of this coded map has been given in recent papers [19,33].

The weight in bytes of the vector file storing the ρ values of the measurements is w(ρ,t). On the other hand, the weight of the coded information produced by wlzip (third column in Table 1) is $w^{*}\left(\rho^{*}\right)$. The mutability of the vector file is the ratio [11,15]:

(3) $$\zeta \left(\rho ,t\right)=\frac{w^{*}\left(\rho^{*}\right)}{w(\rho ,t)}\;.$$

This expression evaluates to 0.944 for the example in Table 1, as shown in its last row.

**Tsallis entropy**. We also include the nonextensive Tsallis entropy by [10,32]:

(4) $$S_{q}=\frac{1}{q-1}\left(1-\sum_{i=1}^{\Omega }p_{i}^{q}\;\right).$$

where *q* is a parameter that indicates the interconnection of the events. Thus, in the limit when q→1.0, the Boltzmann–Gibbs–Shannon entropy is recovered. The probability $p_{i}$ of getting the *i*-*th* value for the observable can be the same as those already obtained in the calculation of Shannon entropy. The sum is over all the states *i* belonging to the cluster of Ω accessible states in the configuration space. This is the complete set of possible states from which the probabilities $p_{i}$ decide which of them are really present.

In [32], Sotolongo-Costa and Posadas propose a statistical model in which, starting from the process of rock fractionation due to the movement of tectonic plates and using Tsallis entropy, they determine the distribution of rock sizes and then, by extremizing the entropic functional of this size distribution, they arrive at an analytical expression for the energy distribution of earthquakes. So, the value of *q* can be obtained from the normal distribution of the values as given by the linear decay in the Gutenberg–Richter law, obtaining an excellent fit of this relationship for different seismic data sets [12,13,32,34,35,36,37], particularly for subduction zones, such as the cases of the Tohoku earthquake in Japan in 2011 [34].

Following the Sotolongo–Posadas approach and above a magnitude threshold, we can relate the corresponding slope *b* to the *q* value by means of the following equation [12,34,35]:

(5) $$b=2\frac{2-q}{q-1}.$$

In general, 1.0≤q≤2.0, but in seismology, as *q* grows, the correlation among seisms also increases and the $S_{q}$ entropy is less additive, which is exactly the reason to consider Tsallis entropy as relevant for the data under consideration (see, for instance, the Appendix A in Flores-Márquez et al. (2024) [37]).

In summary, we deal with three complementary functions: Shannon entropy, Tsallis entropy, and mutability. Shannon entropy is appropriate for dealing with extensive systems characterized by nearly independent or local events. On the contrary, Tsallis entropy copes with non-extensive systems with inter-dependent events and long-range interactions. Both entropies use probabilities without considering the dynamics or the temporal distribution of events. Mutability instead builds a new file or map of the succession of events, keeping track of the dynamics, making it possible to recover the original file from the map. As we will see below, there is a similarity in mutability with Shannon entropy, while the variations of mutability are nearly opposite to those of Tsallis entropy. This last point is reflected in the *q* value obtained from the Gutenberg–Richter analysis: as *q* approaches 2.0, the non-extensivity dominates. We will also appreciate below that these differences are more notorious for inter-event intervals than for magnitudes.

## 4. Results

**Figure 3:**
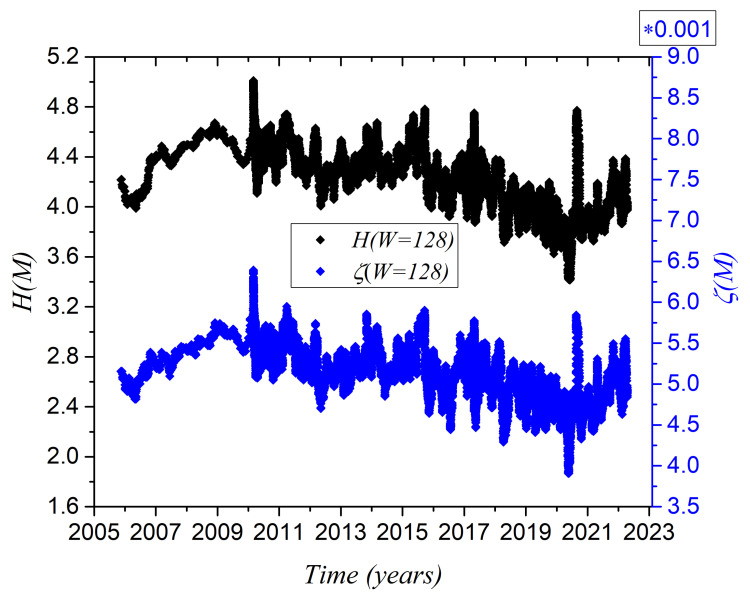
Shannon entropy (*H*) and mutability (ζ) dynamically calculated for magnitude data (*M*) for overlapping windows of 128 consecutive events along the time. The note *0.001 in the upper right means that the values of the right ordinate axis must be multiplied by 0.001 to get the real mutability on the magnitudes.

Figure 3 presents the dynamic results for Shannon entropy and mutability on magnitudes for the time between 1 January 2005, and 12 April 2022. This analysis considers sliding time windows of 128 consecutive events. As with the other dynamic figures, only a single event overlaps the consecutive time windows. Although very similar, these two curves bear subtle differences: consider, for instance, the lower parts of the curves in 2012 or the upper parts of the curves in 2015. So for most of the figures below, we will restrict ourselves to report mutability results only.

Prior to discussing particular details of Figure 3 and others to appear below, it is necessary to interpret the meaning of the peaks in these curves. They are associated with large earthquakes, but they do not directly reflect their magnitudes, which are recognized in Figure 1. Since these functions are associated with entropy, they measure the enlargement of the configuration space available to the system.

Part of this is the large quake itself and its direct aftershock regime, but it may also reflect medium and long-distance effects. Another important factor is the time distribution of these subsequent seisms. At this point, we do not have a single quantitative measure or threshold for Shannon entropy or mutability that captures all these aspects, so we will offer a general description. Lower values of these functions mean repetitive magnitudes (generally low magnitudes). Sharp peaks follow a large earthquake, but the size of the peak does not necessarily escalate with the magnitude (the peaks at 2010 and 2020 are notorious). Several earthquakes occurring close together cause broad maxima. We can confirm this by examining the catalog, which shows this at the beginning of 2011. Drops mean low seismic activity from the point of view of magnitude in Figure 3. Later on, we see that what is a peak for magnitudes is a drop in the corresponding curve for intervals.

To establish whether the structure of the entropic functions corresponds to physical reasons and not mere statistical noise, we have compared the results for the catalog data with the same data after randomization. As reported in Appendix A, which discusses the technical details, the difference is quite notorious. So, the structure of the temporal curves for Shannon entropy and mutability reflects physical properties of the system.

The effect of the $M_{w}$8.8 earthquake is clear in the absolute maxima at the beginning of 2010 in both curves of Figure 3. Visual inspection clearly reveals a distinct behavior prior to the major quake compared to the years afterward. Previously, oscillations were small, and both mutability and Shannon entropy increased in magnitude. Afterwards, oscillations are large, and the general tendency is to decrease. At the end of this period, the system still does not recover the trend previous to 2010.

**Figure 4:**
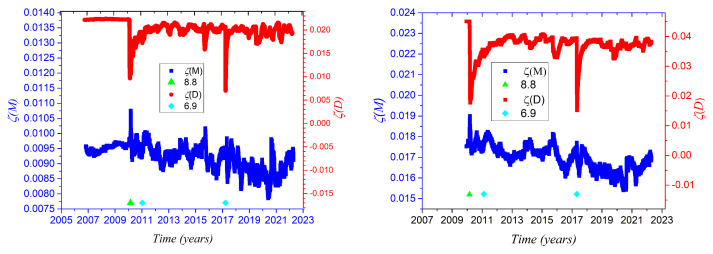
Dynamical mutability for both the magnitude series (lower curves) and the interval series (upper curves). The left panel considers overlapping windows of W=256 consecutive seisms, while the right panel considers W=512 consecutive seisms. Special symbols indicate the time of important earthquakes, as shown in the inset.

We will now focus on inter-event intervals *D*, which we can define as D(i)=t(i)−t(i−1), with *i* as an index through the set of filtered seisms (D(1)=0). Figure 4 shows the mutability corresponding to D(i) in the upper part of the panels. The panel on the left-hand side is for windows of 256 consecutive events, while the panel on the right-hand side is for windows with 512 consecutive events. The insets highlight the symbols marking the times of the three most significant earthquakes during the study period.

The size of the time windows *W* is important, and Appendix B addresses its technical details. We considered different window sizes, with W consecutive events, prior to and after the main $M_{w}$8.8 earthquake, aiming to optimize the difference between results obtained with Shannon entropy and with mutability applied both to magnitudes and to intervals. Short windows mean poor statistics and less reliable results, while long windows improve statistics but separate cause from effect. In Appendix B, we conclude that intervals with W=128 or W=256 yield results that distinguish between regimes. In some cases, time windows can span 64 or 512 consecutive events, since there is no critical threshold.

Including W=128 from Figure 3, we have results for three time windows. Larger windows make the curves less noisy, but they progressively lose information from the quieter period before 2010 (see Figure 1). The three curves show that the magnitude curves reached their absolute minimum in 2020, even though some details were also lost. From there, the curves increase, similar to the period before the 2010 earthquake, but with large oscillations. This area has been active for centuries, so its activity appears to persist, but we cannot predict the intensity or timing of future events.

Mutability of intervals is less noisy than mutability of magnitudes. Large earthquakes, followed by aftershock regimes, cause sharp vertical descents because many earthquakes occur over the following weeks and months. The recovery after a large earthquake is clearer with mutability for intervals than with mutability for magnitudes. However, Figure 4 also shows that the mutability for intervals still does not recover to the levels prior to 2010, confirming the observation already enunciated for the mutability curves on magnitudes. This last point is better observed for time windows with W=512. Actually, the combination of different windows and the consideration of mutability on both magnitudes and intervals makes a battery of tools to better characterize the seismic zone.

**Figure 5:**
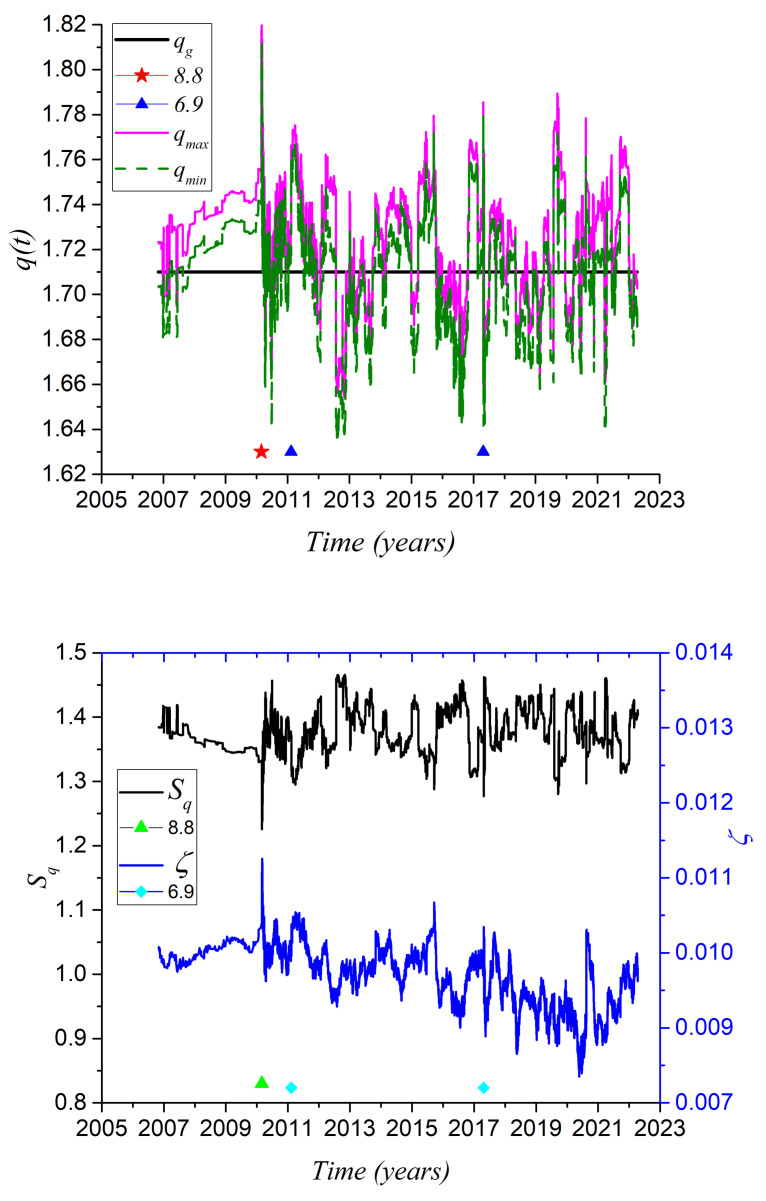
(**Upper panel**): Dynamic behavior of the q(t) parameter got from Equation (4) using a mobile window of 256 consecutive events. The actual q(t) curve is omitted, plotting the upper bound ($q_{max}=q\left(t\right)+\sigma \left(t\right)/2)$, and lower bound ($q_{max}=q\left(t\right)-\sigma \left(t\right)/2)$ bounds, where σ(t) is the dynamical standard deviation. The straight hick line corresponds to the $q_{g}$ value obtained from the global analysis done for the GR analysis presented in Figure 2. (**Lower panel**): Dynamic Tsallis entropy (black curve) and dynamic mutability for magnitudes and W= 256 (same as in Figure 4). Main earthquakes are marked by special symbols as indicated in each inset.

The Gutenberg–Richter analysis on magnitudes presented in Figure 2 leads to a $b=b_{g}=0.82$, which in its own turn yields a value $q_{g}=1.71$, which appears as a horizontal line in the upper panel of Figure 5. The dynamical q(t) function can be obtained using the b(t) function obtained with Equation (5) for the series of time windows with W=256 consecutive events, using the same procedure used to get the global value $b_{g}$ leading to $q_{g}$. To avoid overcrowding, as shown in Figure 5, we do not plot q(t) itself but rather the upper bound ($q_{max}=q\left(t\right)+\sigma \left(t\right)/2)$ and lower bound ($q_{max}=q\left(t\right)-\sigma \left(t\right)/2)$ bounds, where σ(t) is the dynamical standard deviation. The absolute maximum of these curves coincides with the $M_{w}$8.8 earthquake. We observe different variations of these functions comparing prior to the megathrust (small oscillations) and after it (large oscillations).

With the function q(t), we can calculate the dynamical Tsallis entropy by Equation (4). The upper curve represents this in the lower panel of Figure 5. For comparison, we have also plotted here, as the lower curve, the dynamical mutability for W=256. It is interesting to note that these two curves move in opposite directions as they develop. However, $S_{q}$ oscillates around a constant value, while ζ oscillates around a guide that reached a minimum in 2020 and has been recently attempting to recover to the values prior to the $M_{w}$8.8 earthquake. From this point of view, mutability offers more information than the $S_{q}$ entropy.

**Figure 6:**
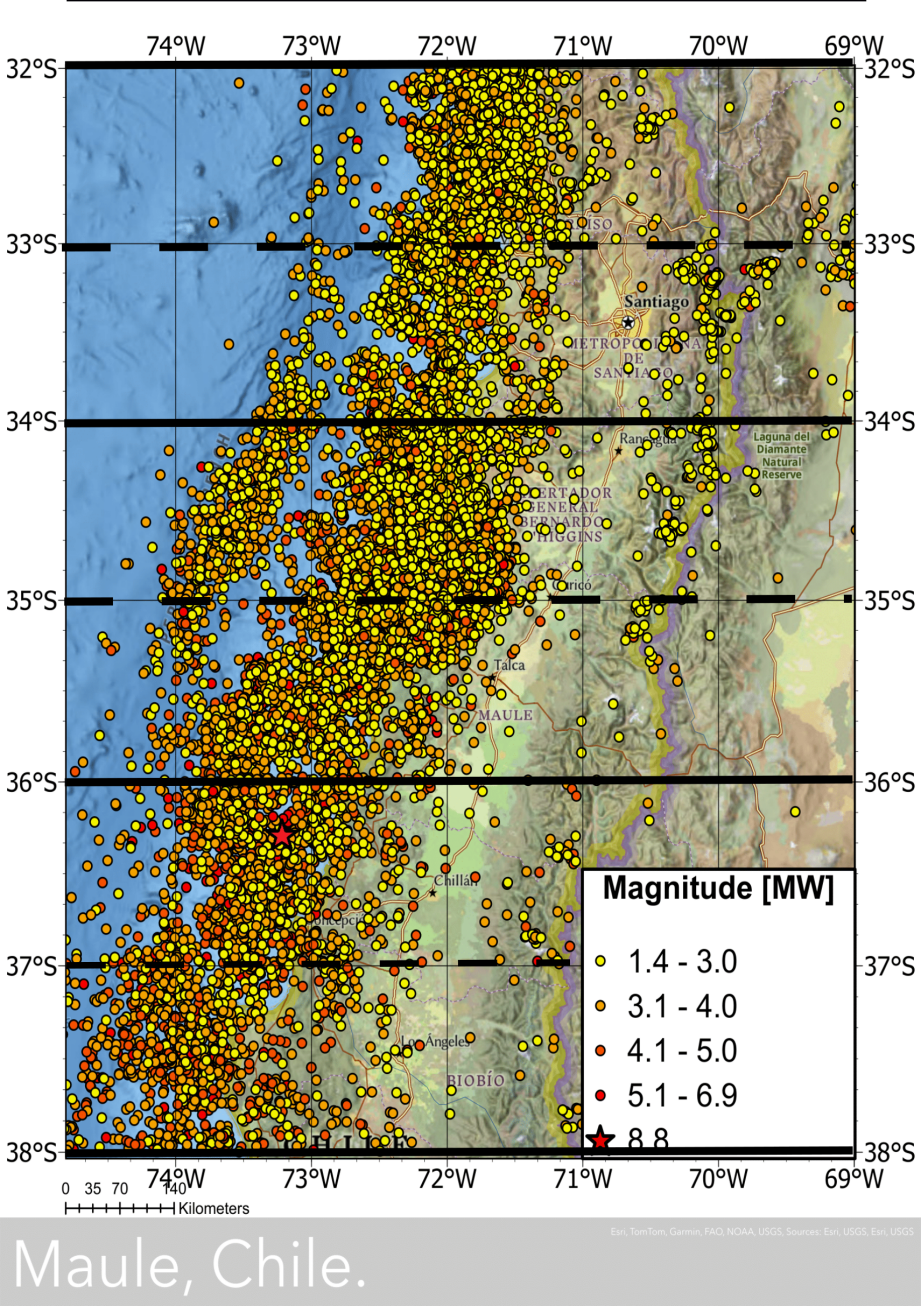
Map of the Maule zone showing the sub-zones of 2 latitude degrees each. The different magnitudes of the seisms are defined in the inset; a red star identifies the 8.8 earthquake.

Figure 6 shows how the zone under study, between parallels 32° S and 38° S, was divided into five overlapping subzones of two degrees each. We shall refer to these subzones as first, second, third, fourth, and fifth, enumerating them from north to south. The number of seisms varies among the subzones. There are 6086, 8900, 8344, 3857, and 2728 seisms for the regions first, second, third, fourth, and fifth, respectively. Within each subzone, we calculated Shannon entropy and mutability.

Let us recall that the subduction trench here is offshore, parallel to the Chilean shoreline, with a north–south orientation; in this way, the subdivision into west–east stripes makes it possible to bring out details from different portions of the trench and the corresponding penetration beneath the Continental Plate. Each degree of latitude spans approximately 110 km, so two degrees corresponds to 220 km, which provides a sufficient number of seismic events for the entropy analysis. The overlap of one degree points to important seismic activity lying near the border between subzones. Since we found in the global analysis that results on intervals were more stable with shorter time windows, we will use inter-event intervals only to discuss results for the subzones.

Because there are fewer earthquakes recorded for the fifth subzone (from 36° S to 38° S), we will report its results separately, in Appendix C at the end of the paper. This subzone (as the fourth subzone) also contained the largest earthquake in this study, so it is important to include its notable entropic manifestations.

**Figure 7:**
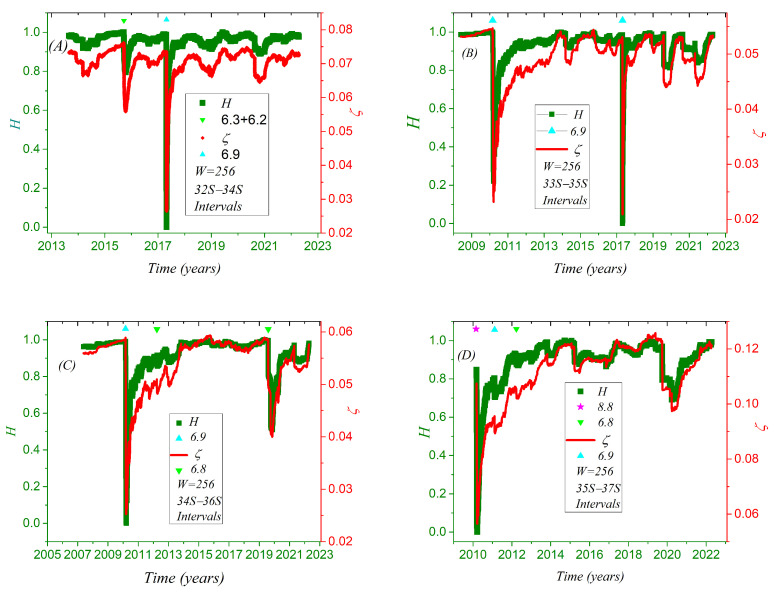
Shannon entropy and mutability for interval sequences. (**A**) 32° S to 34° S; (**B**) 33° S to 35° S; (**C**) 34° S to 36° S; (**D**) 35° S to 37° S. Important earthquakes are marked with special symbols as indicated in the insets.

Figure 7 shows Shannon entropy and mutability on intervals for the northern four sub-zones. The corresponding insets provide the geographic delimiters for each sub-zone, along with the time window W=256 and the most significant earthquakes in each case. Actually, the progressive lack of seismic data from north to south is notorious in the lack of data before 2010 in the last plot.

Mutability and Shannon entropy present similar behavior in the four subzones. We adjusted the ordinate scales to give approximately the same vertical span. This allows us to recognize more details with mutability, as already discussed in the general plots. The largest earthquake in the first sub-zone occurred in 2017, with a magnitude of 6.9, which produced a significant drop in both curves. We do not observe any appreciable difference in either of the two functions before and after this earthquake. The 2010 megathrust produced no significant activity in the first subzone.

The largest event within the second subzone was the same seismic event, $M_{w}$6.9, of the previous subzone (it lies in the overlap between the first and second sub-zones). However, here the aftershock activity originated from the $M_{w}$8.8 earthquake of 2010 is clearly observed. As discussed in the global analysis, the mutability curve provides more information than Shannon entropy; this difference is notable during the aftershock period between 2010 and 2013.

The third sub-zone presents seisms with magnitudes 6.9 and 6.8. However, the most important aftershock response comes from the 2010 earthquake that originated in the following sub-zone. Of secondary importance is the response to the 6.8 quake of 2019.

Finally, the fourth subzone includes the $M_{w}$8.8 earthquake, producing absolute minima in both curves. The aftershock period up to 2014 is more clearly illustrated by the mutability curve. The 6.8 earthquake of 2019 in the third subzone is also present here, and its activity prolonged into 2020 as a swarm of intermediate-magnitude earthquakes. Data is scarce at the beginning of the period, resulting in the loss of both curves prior to the large earthquake. Actually, this effect is even more pronounced in the fifth sub-zone, which is the main reason for omitting such results here. However, we present the analysis for this subzone in Appendix C, but with W=64 only.

Both the general picture and the subzone analysis illustrate that the time series, based both on magnitudes and intervals, carry important information about the seismic characteristics of the region under study. The entropic functions explore the states reached because of the subduction process, allowing us to characterize the different regimes. Thus, before a large seismic event, the dispersion of magnitudes decreases, so Shannon entropy and mutability remain almost constant without large oscillations. At the same time, inter-event intervals grow, being all different, producing large and almost constant values of Shannon entropy and mutability calculated with intervals prior to a major earthquake.

These characteristics can contribute to a battery of indicators aiding in the analysis of tectonic seismic activity. However, other features should also be considered. We mention three independent characteristics that can also be taken into account, if the local conditions allow it. First, the emission of electromagnetic radiation (EMR) produced by the fracture of underground rocks [38,39,40,41,42]. Second, the emission of radon due to ruptures in the porous nature of the crust [43]. Third, the lack of land sliding in subduction zones as measured by Interferometric Synthetic Aperture Radar [44] or GPS monitoring [45]. However, not all these indicators are simultaneously present in any region, as the underground rocks may not be piezoelectric enough to generate EMR, or radioactive radon may not be present in the rocks underneath, or there may not be enough GPS units on the ground to produce the slide gradients. However, seismic waves travel, and different stations detect them, producing catalogs of magnitudes and intervals. Particularly relevant are the interval series that do not depend on a propagation model and can be detected with the precision of seconds. As we saw above, it is precisely the mutability of interval series that is the most sensible indicator, especially in areas where many seismometers are available.

## 5. Conclusions

The subduction zone in front of the Maule region in Chile (from 32° S to 38° S) experienced a large $M_{w}$8.8 earthquake at the beginning of 2010 after decades of calm. The evaluation of the earthquake sequence using entropy functions yields information about the behavior preceding the megathrust and following it.

Shannon entropy presents a minimum 4 years before the major earthquake, gradually increasing without important oscillations. In coincidence with the 8.8 seism, Shannon entropy presents an absolute maximum. From that point on, Shannon entropy had relatively large oscillations, reaching a minimum in 2020. This could mean that the number of states decreased, with only similar magnitude seisms. Because of the distribution in Figure 1, these should correspond to small magnitudes. Then, a seism of medium to large magnitude produces a new increase in the Shannon entropy, which has been increasing afterwards. Mutability presents a similar curve but with better marked oscillations.

When the dynamical window was increased from 128 to 256 and then to 512 consecutive events, the minimum activity during 2020 became more notorious. However, information prior to 2010 is progressively lost, which makes W=256 an appropriate window to dynamically calculate entropy-related functions associated with seismic variables.

Mutability in the inter-event intervals yields complementary information, in a manner opposite to Shannon entropy and mutability of magnitudes. Thus, mutability for intervals is flatter than mutability for magnitudes, meaning that most intervals are different except immediately after a large quake, when the aftershock regime produces many earthquakes at short and similar intervals. The average mutability for intervals remains below the average value before 2010, showing that quakes do not reach the conditions similar to those of the foreshock regime.

Tsallis entropy also varies according to the seismic activity. After a large earthquake, the aftershock regime produces chains of interconnected seisms, which result in increases in the *q* values accompanied by simultaneous decreases in the $S_{q}$ values. However, there are no clear long-run tendencies observed. Eventually, the method of calculating *b* within 256 consecutive events does not allow for better precision. However, larger time windows are not always possible because of the scarcity of data, especially before large earthquakes.

Each subzone presents its own characteristics. The first sub-zone (32–34° S) exhibited significant activity at the beginning of 2017 because of a 6.9 earthquake. However, the aftershock response to the 2010 major earthquake is absent here. On the contrary, this large seismic event is clear in the southernmost sub-zones, from the second to the fifth. This indicates that the rupture traveled north over four sub-zones, approximately 400 km. The nearly constant level of mutability and Shannon entropy prior to 2010 has not recovered yet, which means the subduction process has not clogged again.

Entropy measurements can characterize seismic activity at different locations and times. Although they cannot currently forecast the occurrence of earthquakes, combining these techniques with other complementary indicators could identify regions with higher seismic risk. A combination of high-quality data is necessary to make progress in this direction.

## Figures and Tables

**Table 1:** Column 1 is just an enumeration of measurements; column 2 renders the sequential magnitudes of 32 seisms; column 3 reports the coded information produced by wlzip; column 4 gives the frequency of the value *M* given in boldface at the beginning of the third column; column 5 reports the probability $p_{M}$ of measuring the previous value *M*. The last row shows the mutability value obtained from wlzip.

*i*	*M*	CodedM	$f_{M}$	$p_{M}$
1	2.9	**2.9** 0 8, 2 10 12	5	0.156
2	2.6	**2.6** 1 5 5	3	0.094
3	2.8	**2.8** 3 16 9	3	0.094
4	2.7	**2.7** 4, 2	2	0.063
5	2.7	**2.5** 5 2 5, 2	4	0.125
6	2.5	**3.6** 10 13, 3	4	0.125
7	2.6	**6.8** 14	1	0.031
8	2.5	**4.6** 15	1	0.031
9	2.9	**3.8** 16	1	0.031
10	2.9	**4.0** 17	1	0.031
11	3.6	**3.0** 20	1	0.031
12	2.6	**3.4** 21, 2	2	0.063
13	2.5	**4.5** 26	1	0.031
14	2.5	**5.7** 28	1	0.031
15	6.8	**5.4** 29	1	0.031
16	4.6	**3.1** 30	1	0.031
17	3.8			
18	4.0			
19	2.8			
20	2.9			
21	3.0			
22	3.4			
23	3.4			
24	3.6			
25	3.6			
26	3.6			
27	4.5			
28	2.8			
29	5.7			
30	5.4			
31	3.1			
32	2.9			
ζ		0.944		

**Figure A1:**
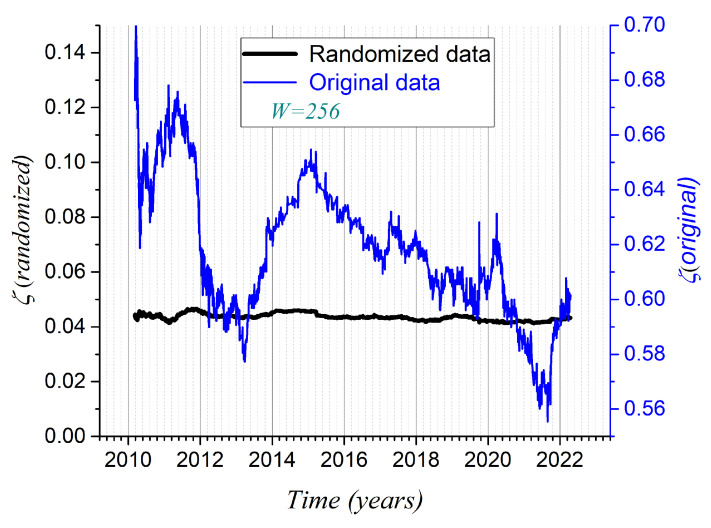
Mutability for the fourth subzone (35–37° S) compared with the mutability for the same magnitude data after randomization.

**Figure A2:**
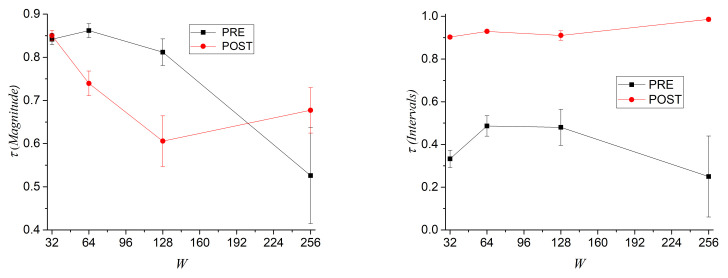
Kendall correlation coefficients for magnitudes (**left**) and for Intervals (**right**) for windows W= 32, 64, 128, and 256. Comparison of the series previous (PRE) and after (POST) the $M_{w}$8.8 earthquake. Mixed behavior for magnitudes, clear difference for intervals mainly for the large windows.

**Figure A3:**
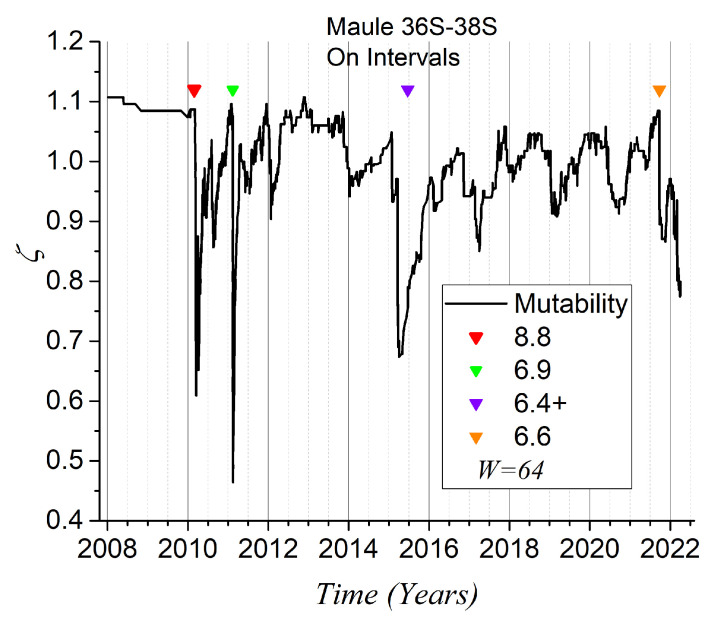
Mutability for the fifth subzone (36–38° S) using a time window of only 64 events because of the lack of enough data in this subzone. The inset defines special symbols that mark important seisms. The broad drop of 2015 corresponds to a sequence of seisms marked as 6.4+.

## Data Availability

The original data presented in the study are openly available in the online site of U. de Chile (2013): Red Sismologica Nacional. International Federation of Digital Seismograph Networks at https://doi.org/10.7914/sn/c1 accessed on 20 October 2023 or URL https://www.fdsn.org/networks/detail/C1/ accessed on 20 October 2023.

## Abbreviations

The following abbreviations are used in this manuscript:

CSN	Centro Sismológico Nacional
GR	Gutenberg–Richter
$b_{g}$	Global *b*-value
$q_{g}$	Global *q* value

## Appendix A

In this Appendix, we compare results for magnitude data from the catalog with the same data after randomizing it. The purpose is to appreciate whether the variations in the data are random noise or correspond to physical reasons.

We will start by describing how the randomization is performed. The starting point is the original file of magnitudes for the fourth subzone, which contains the largest $M_{w}$8.8 earthquake of 2010, with 8344 records. The mutability with a time window of W= 256 is calculated and plotted in blue (thin line) in Figure A1.

We developed our own C++ program to randomize the previous file, and it generates a new file. The program randomly selects two records from the original file, interchanges their positions, and stores them in a new file called R1. This process continues until all records in the original file have been visited. Then, a second randomization follows the same procedure, but it uses file R1 as the original file this time. This process creates an R2 random file for the rest of the analysis.

The random file R2, containing the same records as the original file but as described in the previous paragraph, is now compressed with wlzip using the same time window *W* = 256 as it was done with the original file. The black (thick) line plots the resulting mutability in Figure A1.

Plotting both curves with the same ordinate range allows us to better appreciate their differences. Aside from the different mean value, the original data contains a nontrivial time sequence. Comparing this curve with Figure 7D, which shows mutability for the interval sequence in the same subzone, reveals that the 2010 maximum stems from the $M_{w}$8.8 earthquake, the continuous activity during 2011 is clear, and the 2020 sequence of seismic events is also recognizable.

In summary, natural phenomena can explain the variations presented by the mutability curves for magnitude and intervals, rather than random variations.

## Appendix B

In this Appendix, we conduct an analysis on the window sizes. To do a dynamic analysis of the entropic functions, we need to decide on the time windows on which the statistics can be based. Short windows bear poor information while long windows can mean several months or even years to gather the needed events.

We need an indicator that tells us about the data coverage within the window and that hopefully can differentiate between the regime prior to the main shock and after it. As we have seen in the main text, Shannon entropy and mutability produce similar results, so an appropriate window could also allow us to better compare these two results. To do these tasks, we make use of the Kendall method [46,47]. This method compares different series that measure properties on the same set of data (magnitudes and intervals in our case) by analyzing their variations in time. One series is for Shannon entropies: $\left\{H_{1}^{W},H_{2}^{W},\ldots H_{i}^{W}\ldots ,H_{N}^{W}\right\}$. The other series is for the corresponding mutabilities: $\left\{\zeta_{1}^{W},\zeta_{2}^{W},\ldots \zeta_{i}^{W}\ldots ,\zeta_{N}^{W}\right\}$. Both series were obtained for the same window *W* and have the same number of N elements. Then, within each series, we compare consecutive values and count variations, which leads to a Kendall correlation coefficient whose usual symbol is τ. At this point, we omit technical details that would need more than an appendix and would lead us to other fields of study; however, the interested reader can follow this line of thought through the already quoted references.

In addition, we will separate the analysis in two parts: (i) with data prior to the main earthquake $M_{w}$8.8, and (ii) with data beginning with the $M_{w}$8.8 seism to the end of the series. Since τ is a normalized coefficient, we can compare the tendencies before and after this quake.

In Figure A2, we present the Kendall indicator for magnitudes and intervals for windows of 32, 64, 128, and 256 elements for the entire data set. Squares (circles) represent the value of the Kendall coefficient for the data previous (after) the $M_{w}$8.8 earthquake. Error bars are also included.

As it follows from this figure, the Kendall correlation coefficient for magnitudes PRE decreases with the time window preventing for using *W* larger than 256 say. In the same period of time, POST results tend to stabilize in the range of windows with 128 and 256 events. Error bars increase with *W*. On the other hand, results for intervals are more stable, especially for POST results that are almost constant and saturated close to unity with tiny error bars. PRE results for intervals are lower with a tendency to decrease with *W*, with increasing error bars.

The difference between PRE and POST results for τ is nearly nil in the case of magnitudes for W=32 and clearly discriminates for W=128. This is another reason to avoid small windows. In the case of intervals the difference is present for all window sizes maximizing at W=256. As already pointed out, large windows face a practical disadvantage: the cause and effect may fall apart for long periods of time, especially with the scarce activity prior to a large earthquake. An example of this last situation occurs in the Figure A3 corresponding to the next Appendix C, where we needed to use W=64, otherwise the main event would be hidden within the interval.

For all the reasons given above, we will prefer W=128 or W=256 in most of the analyses, but without sticking firmly to one of them, since there are slight advantages and disadvantages for any of them.

## Appendix C

In this Appendix, we present the mutability results for the case of the fifth subzone, 36–38° S. This figure was not included along with the others of Figure 7 since the largest possible time window is only W=64 due to the fewer data available for this subzone, which is 2728 data points.

Since we showed in the previous appendix that intervals discriminate better between regimes previous and post the major shock, we present results on mutability for intervals only in Figure A3. Before the $M_{w}$8.8 earthquake, mutability remains nearly flat, as depicted in Figure 7B,C. It should be noted that this behavior cannot be appreciated from Figure 7D since there the lack of data before the major shock is evident.

A double drop revealed the major shock of 27 February 2010 because an important aftershock earthquake occurred soon afterwards. Then, an isolated earthquake of magnitude 6.9, at the beginning of 2011, produced the largest drop in this plot, showing that the size of drops and peaks is not a measure of the magnitudes, but rather of the configuration space. This receives further confirmation with the broad drop in 2015, the product of a sequence of intermediate earthquakes maximizing with the 6.4 event marked in the figure. This sequence of seismic events is present in Figure 7D in a weaker form, showing its continuation to the north. Finally, a 6.6 earthquake in 2021 also produced a moderate drop in the mutability curve.

The post-shock activity differs from prior observations before the $M_{w}$8.8, showing that the subduction remained active for at least the 12 years that followed.
